# Successful resection of pancreatic metastasis from oesophageal squamous cell carcinoma: a case report and review of the literature

**DOI:** 10.1186/s12885-019-5549-9

**Published:** 2019-04-05

**Authors:** Wataru Koizumi, Minoru Kitago, Masahiro Shinoda, Hiroshi Yagi, Yuta Abe, Go Oshima, Shutaro Hori, Kenta Inomata, Hirofumi Kawakubo, Miho Kawaida, Yuko Kitagawa

**Affiliations:** 1 Department of Surgery, Keio University School of Medicine, 35 Shinanomachi, Shinjuku-ku, Tokyo, 160-8582 Japan; 2 Department of Pathology, Keio University School of Medicine, Tokyo, Japan

**Keywords:** Isolated metastasis, Pancreas, Pancreatic metastasis, Oesophageal cancer

## Abstract

**Background:**

Oesophageal cancer has a high metastatic potential and poor prognosis, with a significant risk of recurrence after radical resection. However, resected pancreatic metastasis from oesophageal cancer is rare.

**Case presentation:**

Eleven years prior, a seventy-year-old woman had been treated with transthoracic radical oesophagectomy for oesophageal squamous cell carcinoma. Four years prior, she had undergone chemotherapy for lymph node recurrence at the splenic hilum and achieved a partial response. She had also received chemoradiotherapy for lymph node recurrence at the splenic hilum 3 years prior; a complete response was achieved. However, routine follow-up with abdominal computed tomography recently revealed a tumour at the pancreatic tail and swollen lymph nodes. The patient underwent distal pancreatectomy on the basis of a pre-operative diagnosis of primary pancreatic cancer, although a histological examination of the surgical specimen revealed metastatic squamous cell carcinoma that was compatible with metachronous pancreatic metastasis from oesophageal squamous cell carcinoma. The patient has been undergoing clinical follow-up without adjuvant therapy and has been disease-free for 24 months after resection of the pancreatic metastasis.

**Conclusions:**

Resection of pancreatic metastasis may improve prognosis and should be considered when treating patients with solitary metastasis from oesophageal squamous cell carcinoma.

## Background

Oesophageal cancer has a high metastatic potential and poor prognosis, although recent advances in multimodal treatment using oesophagectomy and definitive chemoradiotherapy or chemotherapy have improved patient outcomes [1]. However, recurrence after radical oesophagectomy is detected in 30–50% of patients with haematogenous or lymphatic recurrence [2–4]. Pancreatic metastasis from oesophageal cancer is rare, with frequencies of 0.1, 0.7, and 2.9% for oesophageal cancer, metastatic oesophageal cancer, and oesophageal squamous cell carcinoma (OSCC), respectively [5]. Furthermore, less than 5% of cases of pancreatic metastases involve oesophageal cancer [6–8], and only few reports in the English literature have described the resection of pancreatic metastases from oesophageal cancer [9–11]. Here, we report a case of metachronous pancreatic metastasis from oesophageal cancer and provide a review of the literature.

## Case presentation

**Fig. 1:**
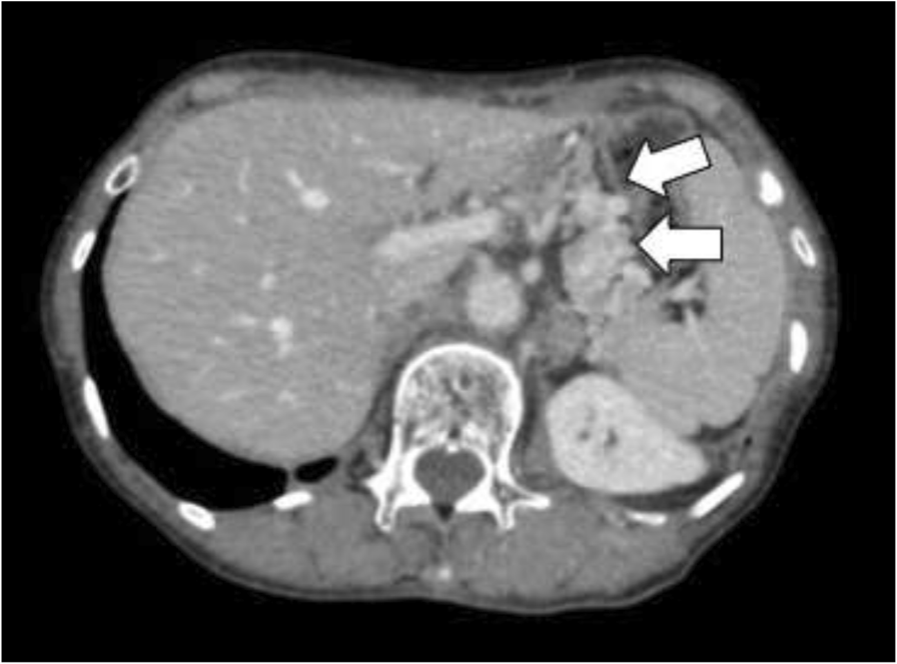
Lymph node recurrence. Lymph node recurrence (white arrows) was detected at the splenic hilum 1 year after chemotherapy

**Fig. 2:**
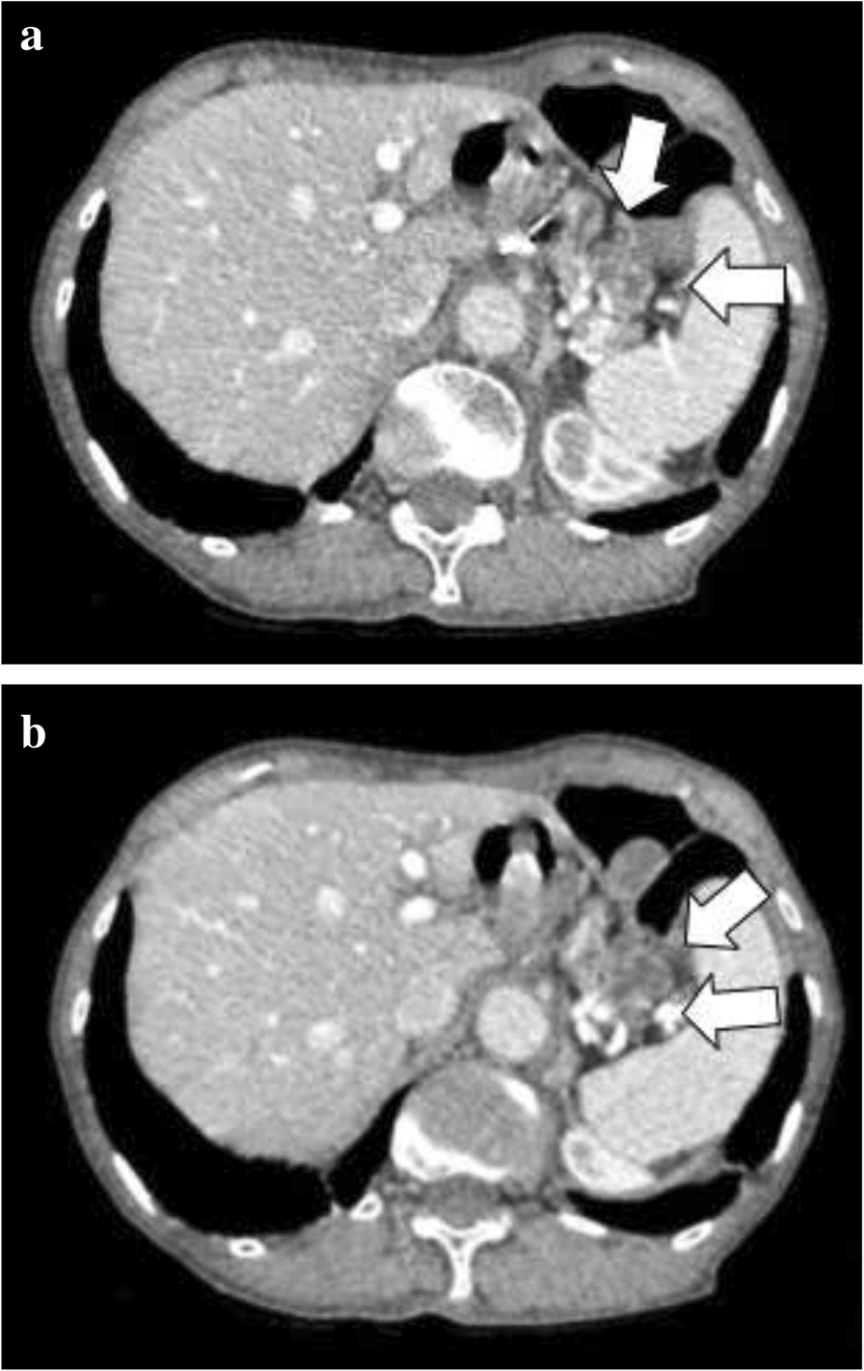
Pre-operative computed tomography findings. Pre-operative computed tomography imaging detected **a** an adherent mass at the pancreatic tail and **b** a tumour above the mass

**Fig. 3:**
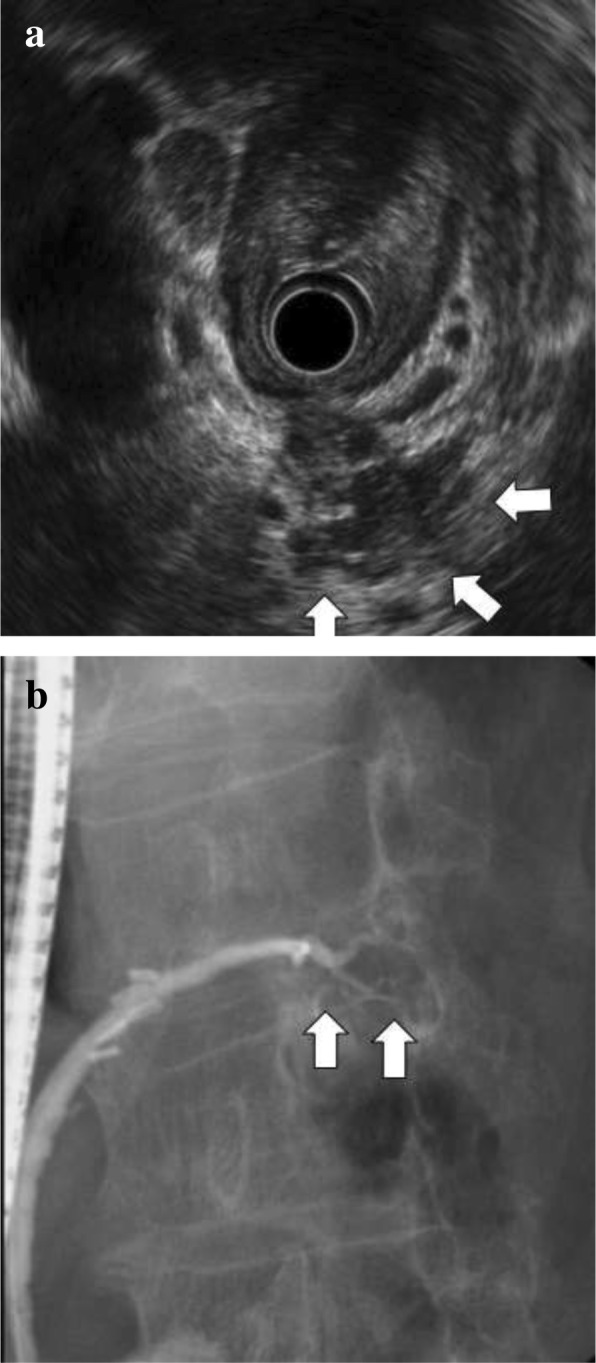
**a** Pre-operative endoscopic ultrasonography/retrograde pancreatography findings. Pre-operative endoscopic ultrasonography detected an adherent mass at the pancreatic tail; **b** endoscopic retrograde pancreatography revealed narrowing of the main pancreatic duct in the pancreatic tail

**Fig. 4:**
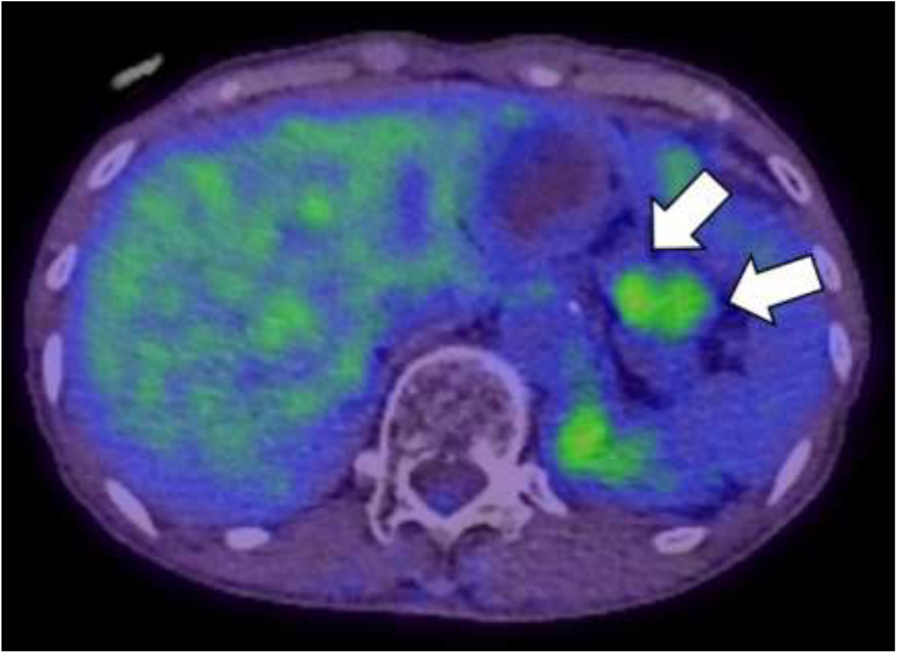
Positron emission tomography/computed tomography findings. Positron emission tomography/computed tomography revealed a high standardised uptake value of 4.69 in the pancreatic tail mass (white arrows)

**Fig. 5:**
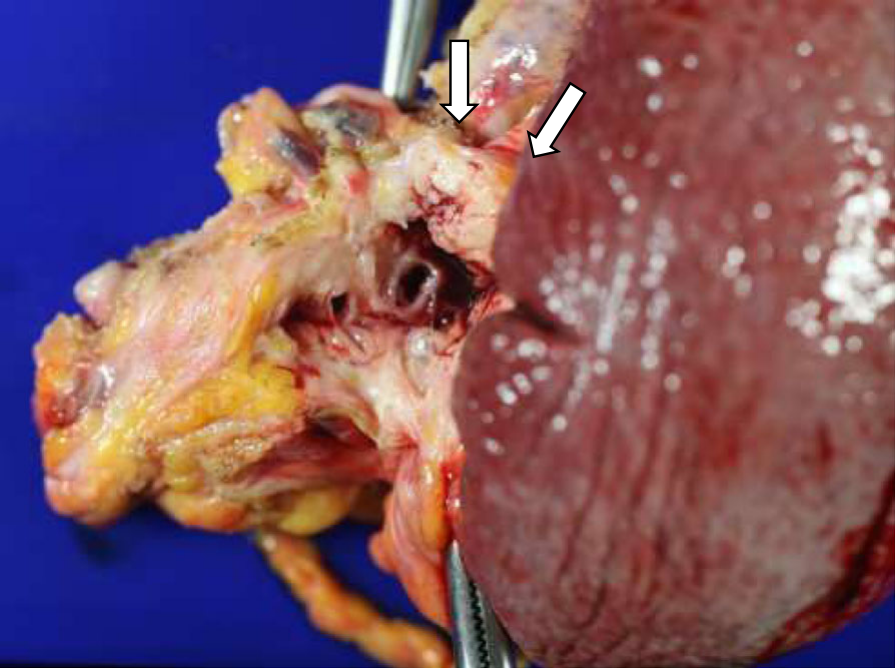
Macroscopic examination. Macroscopic evaluation revealed a whitish tumour (measuring 29 × 22 × 30 mm) in the pancreatic tail

**Fig. 6:**
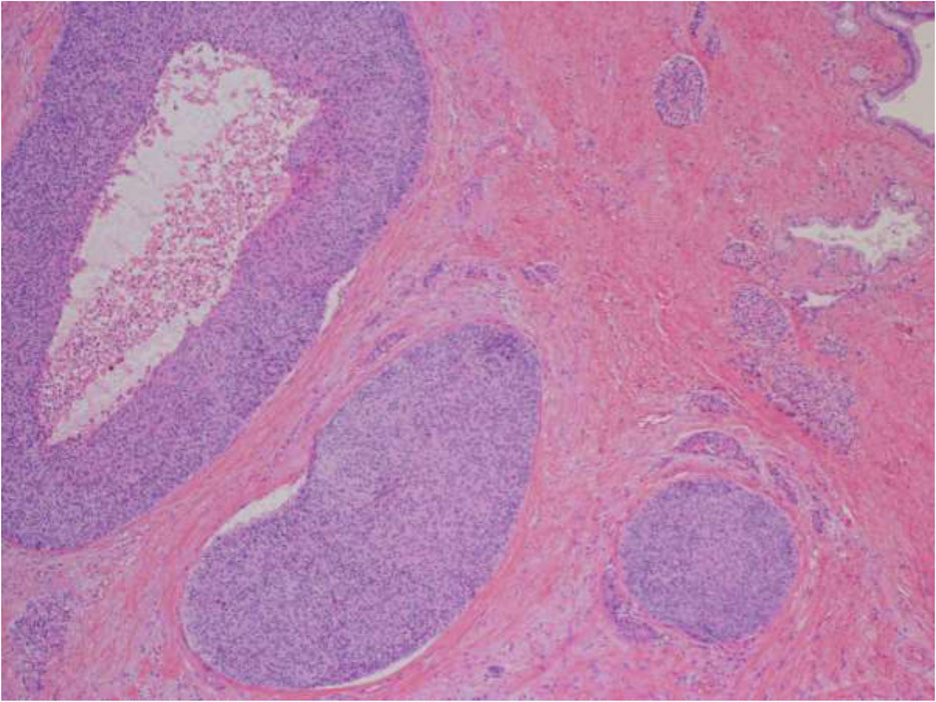
Microscopic examination. Microscopic examination of squamous cells resembling oesophageal carcinoma resected 11 years prior showed that the pancreas exhibited a fibrous change in the background of the carcinoma

Eleven years prior a seventy-year-old woman had undergone transthoracic radical oesophagectomy and cervical lymphadenectomy for OSCC (pathological T1bN0M0 stage I, according to the 7th edition of the Union for International Cancer Control/American Joint Committee on Cancer staging system). Seven years after surgery, computed tomography (CT) revealed lymph node recurrence at the splenic hilum, which was treated with four courses of cisplatin plus 5-fluorouracil. Treatment reduced the size of the lymph node, although 1 year later, lymph node metastasis was detected again at the pancreatic tail and splenic hilum (Fig. 1). Chemoradiotherapy (50 Gy in 28 fractions) resulted in a complete response at the lymph nodes. However, 11 years after surgery, a cystic solid tumour was detected at the pancreatic tail using CT (Fig. 2a–b) and endoscopic ultrasonography (Fig. 3a). A change in the main pancreatic duct was also detected using endoscopic retrograde pancreatography (Fig. 3b). No other metastases were seen on evaluation with positron emission tomography/CT (Fig. 4), which strongly suggested primary pancreatic tail cancer with lymph node metastasis. Intraductal papillary mucinous carcinoma was one of the differential diagnoses that were considered. Distal pancreatectomy and splenectomy with lymphadenectomy were performed to treat the tumour, which had a diameter of 30 mm and appeared to invade the artery of the gastric tube that had been reconstructed during the oesophagectomy. Thus, the artery was also resected to achieve curative resection. Operative time was 190 min and blood loss was 30 mL. The resected specimen from the pancreatic tail measured 29 × 22 × 30 mm (Fig. 5). Pathological examination revealed that the tumour was a squamous cell carcinoma, which was compatible with the detection of the previous OSCC. The final diagnosis was pancreatic metastasis from OSCC (Fig. 6). The resected metastatic lymph nodes at the splenic hilum also exhibited squamous cell carcinoma cells. The patient was discharged after an uneventful recovery. She is continuing clinical follow-up without adjuvant therapy and has been disease-free for 24 months after resection of the pancreatic metastasis.

## Discussion and conclusions

Oesophageal cancer has a high metastatic potential and poor prognosis. Approximately a fifth of patients have distant metastasis at presentation. These distant metastases most commonly involve the abdominal lymph nodes (45.0%), liver (35.0%), lung (20.0%), cervical or supraclavicular lymph nodes (18.0%), bone (9.0%), adrenal gland (5.0%), peritoneum (2.0%), or brain (2.0%), as well as the stomach, pancreas, pericardium, and spleen (each 0.7%) [5]. Autopsy studies revealed that the incidence of pancreatic metastasis ranged from 1.6 to 5.9% [6, 12]. In this context, pancreatic metastasis consists of a secondary pancreatic tumour or intrapancreatic metastasis [13], with the common sites of the primary tumour being the stomach, lung, extrahepatic bile duct, haematopoietic system, or gallbladder [12]. Pancreatic metastasis from oesophageal cancer is rare, with frequencies of 0.1, 0.7, and 2.9% for oesophageal cancer, metastatic oesophageal cancer, and OSCC, respectively [5]; OSCC is only involved in 0–4.9% of cases of pancreatic metastases [6–8].

To the best of our knowledge, the English literature only contains 3 reports on the resection of isolated pancreatic metastases from OSCC (Table 1) [9–11]. Three cases, including the present case, were metachronously diagnosed during routine CT follow-up, whereas the remaining case was synchronously diagnosed during pre-operative examination for the treatment of primary oesophageal carcinoma. The interval between the diagnosis of the primary oesophageal cancer and the isolated pancreatic metastasis ranged from 0 to 132 months, with our case having the longest interval. In all cases, the pre-operative diagnosis was primary pancreatic carcinoma, and distal pancreatectomy was successfully performed with no surgical complications. A total of 3 reported cases involved planned adjuvant chemotherapy, and the patients were alive without recurrence 4–24 months after surgery.

**Table 1:** Reported cases of pancreatic metastasis from oesophageal carcinoma

Authors (year of publication)	Patient age (years)	Sex	Synchronous/metachronous	Interval between metastases (months)	Surgery	Adjuvant therapy	Follow-up (months)	Recurrence
Esfehani et al. (2011)	59	F	Metachronous	48	DP	5-FU	4	No
Park et al. (2013)	58	M	Synchronous	0	DP	FP	6	No
Okamoto et al. (2014)	68	M	Metachronous	32	DP	FP	9	No
Present case	81	F	Metachronous	132	DP	No	24	No

Abbreviations: *5-FU* 5-fluorouracil, *DP* Distal pancreatectomy, *F* Female, *FP* 5-fluorouracil plus cisplatin, *M* Male

It is important to determine whether pancreatic metastasectomy is an effective treatment option for cases of OSCC. Several reports have indicated that pancreatic metastasectomy provides a favourable prognosis [7, 13, 14], although its effectiveness is dependent on the biology of the primary cancer and whether the metastasis is synchronous or metachronous. For example, long-term survival can be predicted on the basis of the cancer type such as renal cell carcinoma, colon cancer, or sarcoma [7]. Among patients with metachronous metastasis, a poor prognosis is associated with the presence of symptoms at diagnosis, synchronous metastases, and a disease-free interval of < 2 years. Reddy et al. [7] reported that patients may benefit from pancreatic metastasectomy if they fulfil the following criteria: (1) primary cancer type that is associated with a favourable outcome, (2) control of the primary cancer, (3) isolated metastasis that can be resected, and (4) ability to tolerate pancreatectomy. Although the present case involved OSCC, the patient had metachronous disease with a long disease-free interval, unifocal metastasis, and response to systemic therapy, predicting a favourable outcome.

In conclusion, we describe a rare case of pancreatic metastasis from OSCC. On the basis of our findings and a review of the literature, we suggest that pancreatic metastasectomy may be a useful treatment option for isolated pancreatic metastasis from OSCC. However, accumulating evidence from additional cases is necessary to confirm our findings.

## Abbreviations

CT: computed tomography
OSCC: oesophageal squamous cell carcinoma
